# Awareness and Knowledge of Tooth Reimplantation After Avulsion Among Dental and Non-dental Populations: A Systematic Review

**DOI:** 10.7759/cureus.99917

**Published:** 2025-12-23

**Authors:** Abdullah Alshubat, Faris S Bantan, Yasmeen AlNasser, Mashael T Alhejji, Saja H Alsuwailem, Mariya I Alibrahim, Heba A AlkhairAllah, Mona S Alharbi, Faisal F Alrashedi, Dalal Alturaif, Laila Alanaz

**Affiliations:** 1 Pediatric Dentistry, Ministry of Health Holdings, Hofuf, SAU; 2 Dentistry, Riyadh Elm University, Riyadh, SAU; 3 Oral Medicine, Imam Abdulrahman Bin Faisal University, Dammam, SAU; 4 Dentistry, Imam Abdulrahman Bin Faisal University, Dammam, SAU; 5 Dentistry, Qassim University, Buraydah, SAU; 6 Dentistry, Mustaqbal University, Buraydah, SAU; 7 Dentistry, Jouf University, Sakakah, SAU; 8 Restorative Dentistry, Private Practice, Riyadh, SAU; 9 Dentistry, Private Practice, Riyadh, SAU

**Keywords:** awareness, dental trauma, first aid, knowledge, public education, tooth avulsion, tooth reimplantation

## Abstract

Tooth avulsion is one of the most severe forms of traumatic dental injury, and its prognosis depends heavily on immediate and appropriate first-aid management, including prompt reimplantation or correct temporary storage of the avulsed tooth. This systematic review evaluates global awareness and knowledge of tooth reimplantation across dental populations (dentists, dental professionals, and dental students) and non-dental populations (parents, school teachers, emergency physicians, first responders, and the general public). A comprehensive search of PubMed, ScienceDirect, Cochrane Library, and Google Scholar identified 8,133 records, of which 19 cross-sectional studies met the eligibility criteria, representing 5,752 participants from 10 countries. Most studies demonstrated moderate methodological quality. The findings reveal substantial deficiencies in knowledge, particularly among non-dental populations. Awareness that an avulsed tooth can be replanted ranged widely, from as low as 23% among parents to over 80% among dental professionals; however, even among trained providers, detailed protocol-based knowledge was inconsistent. Understanding of critical steps - including the optimal replantation window (<30 minutes), appropriate storage media such as milk or saline, and correct handling techniques - was generally poor across most groups. Only 2-37% of respondents in non-dental populations indicated willingness or ability to attempt immediate replantation. Misconceptions regarding inappropriate storage materials, delayed referral, and handling of the root surface were prevalent. Studies that included educational interventions demonstrated marked improvements in awareness and decision-making. Overall, this review demonstrates a significant global knowledge gap in the emergency management of tooth avulsion, particularly among non-dental populations, underscoring the urgent need for targeted educational interventions and improved dissemination of evidence-based guidelines to enhance reimplantation outcomes.

## Introduction and background

Traumatic dental injuries (TDIs) represent a significant public health concern, particularly among children and adolescents, due to their potential for functional, aesthetic, and psychological consequences. It is estimated that up to one-quarter of school-aged children and one-third of adults may experience some form of TDI [1]. One of the most severe forms of TDI is tooth avulsion, defined as the complete displacement of a tooth from its socket. The prevalence of avulsion has been reported to range from approximately 0.5% to 16% of all dental injuries in the permanent dentition [2]. Avulsion of a permanent tooth is a dental emergency that requires immediate and proper management to optimize the chances of favorable outcomes. It is important to note that avulsion of primary teeth does not require reimplantation and is generally not recommended due to the risk of damage to the developing permanent tooth germ. The prognosis is critically dependent on two key factors handled at the emergency site: the extra-oral dry time (the period the tooth spends outside the mouth) and the choice of storage medium [3]. According to the International Association of Dental Traumatology (IADT) guidelines, prompt reimplantation or appropriate interim storage of the avulsed tooth is essential to preserve periodontal ligament viability and reduce the risk of complications such as ankylosis, inflammatory root resorption, and eventual tooth loss [4]. Hank's Balanced Salt Solution (HBSS), an isotonic, pH-balanced fluid, is recognized as the ideal storage medium for this purpose [3]. Despite clear evidence and established protocols, studies consistently show that adherence to recommended practices remains variable and often suboptimal. For example, a recent scoping review found that, even when clinicians followed IADT-based protocols, long-term survival of replanted teeth still ranged between 55% and 96% [5]. In addition to the technical management by dental professionals, the success of reimplantation is heavily influenced by early first-aid actions by laypersons or first responders at the site of injury. However, evidence indicates that non-dental populations, such as teachers, sports coaches, and parents, often lack adequate awareness and readiness to execute correct emergency measures [6]. Compounding this knowledge gap, the public's first-aid understanding is frequently undermined by dental misinformation prevalent on social media and other informal channels, which can lead to harmful decisions [7]. Conversely, structured educational interventions have demonstrated marked effectiveness [8]. A systematic review of school-based interventions found that, prior to training, educators demonstrated poor knowledge of avulsed tooth management and storage media, which adversely affects prognosis [6]. Similarly, among dental interns, although a high proportion recognize that avulsion is an emergency, fewer are familiar with the ideal transport medium or the critical extra-oral time window for reimplantation [9]. Given these gaps, it is vital to assess the existing awareness and knowledge of tooth reimplantation among various stakeholders - including dental professionals, students, and the wider public. A systematic synthesis of the literature can clarify the current state of knowledge, identify prevailing deficiencies, and inform targeted educational interventions. Accordingly, this systematic review aims to evaluate the level of awareness and knowledge regarding tooth reimplantation after avulsion across different population groups globally, to determine common deficits and propose recommendations for improvement.

## Review

Methodology

Study Design

This study was conducted as a systematic review following the Preferred Reporting Items for Systematic Reviews and Meta-Analyses (PRISMA) 2020 guidelines [10]. The primary objective was to evaluate and synthesize existing evidence on the awareness and knowledge of tooth reimplantation after avulsion among various populations, including dental professionals, medical practitioners, students, teachers, parents, and the general public. The research question and eligibility of this systematic review were structured according to the PICO framework (Table 1).

**Table 1:** PICO framework for the systematic review

PICO Component	Description
Population (P)	Dental populations: dentists, dental professionals, and dental students; Non-dental populations: parents, school teachers, emergency physicians, first responders, and members of the general public
Intervention/Exposure (I)	Exposure to knowledge or awareness regarding emergency management and reimplantation of avulsed permanent teeth
Comparison (C)	Comparison between dental and non-dental populations; and where applicable, between trained and untrained or educated versus non-educated groups
Outcome (O)	Level of awareness and knowledge regarding tooth reimplantation after avulsion, including immediate replantation, appropriate storage media, extra-oral time, and correct handling techniques

Search Strategy

A comprehensive electronic search was conducted in PubMed, ScienceDirect, Cochrane Library, and Google Scholar. The search covered all relevant studies published from inception up to October 2025, with no restrictions on country or language. The search strategy combined Medical Subject Headings (MeSH) and free-text terms using Boolean operators. Key MeSH terms included “Tooth Avulsion,” “Dental Trauma”, “Tooth Replantation”, “Knowledge”, “Awareness”, and “First Aid”. These were combined with keywords such as avulsed tooth, reimplantation, dental injury, emergency management, parents, teachers, dentists, and healthcare professionals. The detailed search strategies for each database are provided in the Appendix. Reference lists of included studies were manually screened to identify additional relevant articles.

Inclusion Criteria

Studies were eligible for inclusion if they employed a cross-sectional, observational, or survey-based design and assessed awareness, knowledge, or attitudes related to tooth avulsion or reimplantation. Included studies involved dental populations - defined as dentists, dental professionals, or dental students - or non-dental populations, including parents, school teachers, emergency physicians, first responders, or members of the general public. Only studies reporting outcomes relevant to emergency management of avulsed permanent teeth, such as immediate replantation, storage media, extra-oral time, or handling techniques, were considered. Peer-reviewed articles published in the English language, with no restrictions on country of origin, were eligible for inclusion.

Exclusion Criteria

Studies were excluded if they were case reports, case series, editorials, commentaries, conference abstracts, or review articles. Research focusing exclusively on clinical procedures, laboratory experiments, animal models, or treatment outcomes without assessing knowledge or awareness was also excluded. Additionally, studies that did not report data relevant to tooth reimplantation or emergency management of avulsion, or those lacking sufficient methodological detail to allow appraisal, were not considered for inclusion.

Study Selection

**Figure 1:**
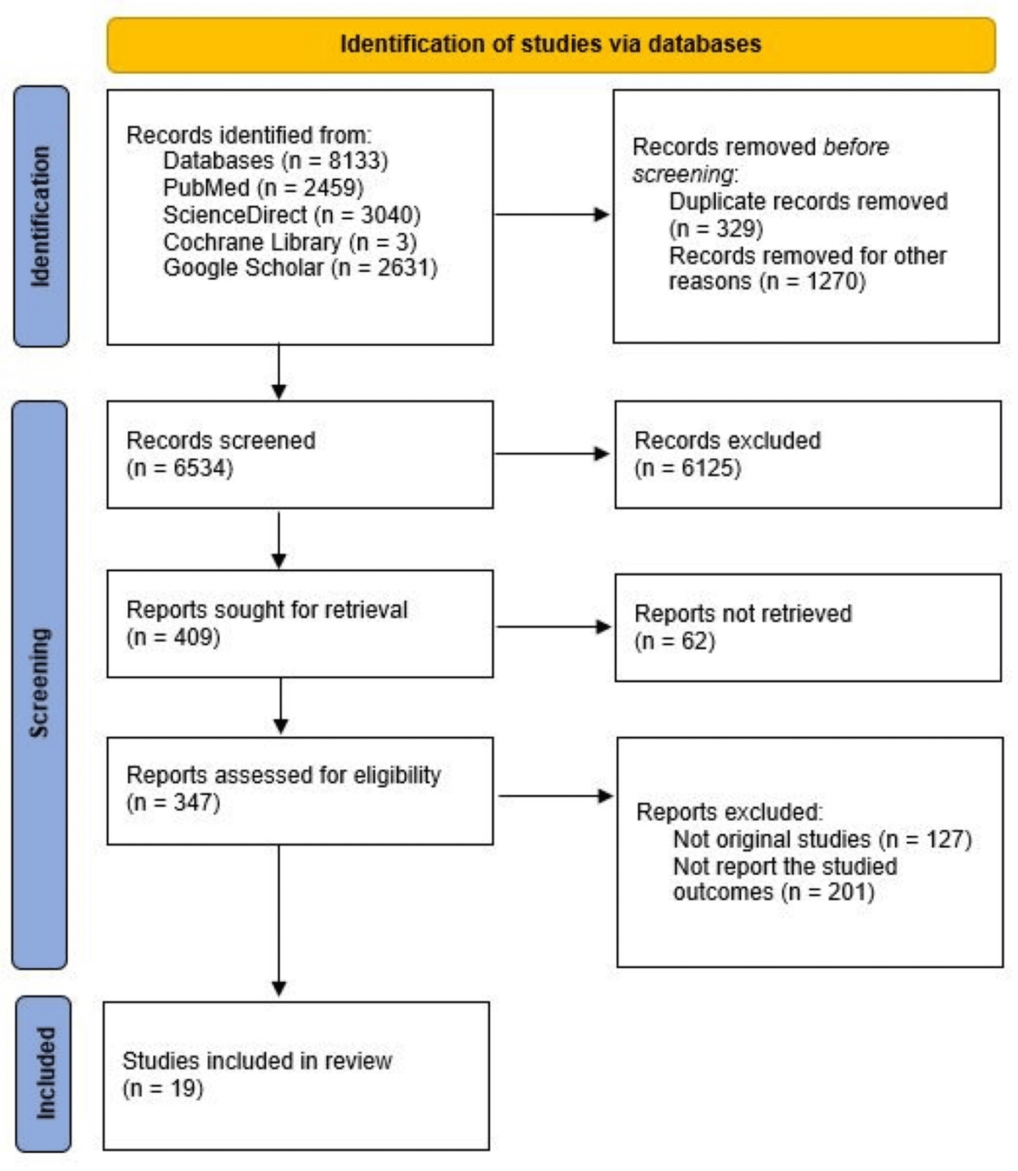
PRISMA flowchart showing the study selection process PRISMA: Preferred Reporting Items for Systematic Reviews and Meta-Analyses

All records were imported into Zotero reference management software for duplicate removal and screening. Title and abstract screening, followed by full-text assessment, was performed independently by two reviewers in a blinded manner. Reviewers were unaware of each other’s decisions during screening. Any discrepancies were resolved through discussion or consultation with a third reviewer. The selection process is summarized in a PRISMA flow diagram (Figure 1).

Data Extraction

A standardized data extraction form was used to collect relevant information from the included studies. Extracted data included author(s), publication year, country, study population, sample size, study design, data collection method, assessment tool or questionnaire used, main findings regarding knowledge or awareness of tooth reimplantation, and key conclusions. Data extraction was carried out independently by two reviewers to minimize errors and bias.

Quality Assessment

The methodological quality of the included studies was evaluated using a modified version of the Newcastle-Ottawa Scale (NOS) for cross-sectional studies [11]. The tool assessed each study based on three domains: selection of participants, comparability of study groups, and assessment of outcomes. Each domain was rated, and studies were categorized as having low, moderate, or high risk of bias according to the total score. Any disagreements between reviewers during quality assessment were resolved through consensus.

Meta-Analysis

A quantitative meta-analysis was not undertaken due to substantial clinical and methodological heterogeneity among the included studies. Considerable variation existed in study populations (dental versus non-dental groups), outcome definitions (awareness, knowledge, attitudes, or willingness to reimplant), assessment instruments, and reporting formats. In addition, studies differed in their definitions of key variables, including extra-oral time thresholds, acceptable storage media, and handling techniques, and frequently reported outcomes as descriptive proportions without standardized effect measures. This heterogeneity precluded meaningful statistical pooling and reliable estimation of summary effects. Although reporting proportions with 95% confidence intervals could aid interpretation, the inconsistency in outcome categorization and data presentation across studies limited comparability. The decision to perform a narrative synthesis rather than a meta-analysis was therefore made as it was methodologically appropriate to avoid potentially misleading pooled estimates. Therefore, findings are presented descriptively and comparatively in accordance with PRISMA recommendations for systematic reviews without meta-analysis.

Data Synthesis

Given the heterogeneity in study designs, populations, and outcome measures, findings were synthesized narratively. The results were organized according to study characteristics, target population, and main outcomes related to knowledge and awareness of tooth reimplantation. Patterns, trends, and regional differences in awareness levels were identified and discussed in the context of existing literature.

Results

Study Selection and Characteristics

The initial search across five databases yielded 8,133 records, of which 329 duplicates and 1,270 irrelevant titles were removed prior to screening. A total of 6,534 records were screened, and 6,125 were excluded after title and abstract review for not meeting the inclusion criteria. Of the 409 full-text articles assessed, 62 could not be retrieved, and 328 were excluded for not being original studies or not reporting the targeted outcomes. Ultimately, 19 cross-sectional studies [12-30] were included in the qualitative synthesis, encompassing 5,752 participants from ten countries - Brazil, India, Morocco, Pakistan, Qatar, Saudi Arabia, Turkey, Yemen, Kuwait, and Oman.

The methodological quality of the included studies, assessed using the modified NOS, ranged from 5 to 8 out of 10, indicating predominantly moderate methodological quality. Most studies demonstrated clear objectives and appropriate participant selection but were limited by the use of convenience sampling and reliance on self-reported questionnaires, which increased the risk of information bias. Overall, six studies were rated as high quality, while the remaining 13 studies were classified as moderate quality, with no study meeting criteria for low risk of bias across all domains (Table 2).

**Table 2:** Quality assessment of the included studies (modified Newcastle-Ottawa Scale)

Study (Author, Year)	Selection (0–4)	Comparability (0–2)	Outcome Assessment (0–4)	Total Score (0–10)	Quality Rating
Al-Asfour et al. (2008) [30]	3	1	3	7	High
Al Barkhati et al. (2023) [13]	3	1	3	7	High
Al-Khalifa et al. (2022) [14]	3	1	2	6	Moderate
Al Sheeb et al. (2023) [15]	3	1	2	6	Moderate
Al-Huthaifi et al. (2025) [16]	4	1	3	8	High
AlGhamdi et al. (2016) [17]	2	1	2	5	Moderate
Alyahya et al. (2018) [18]	3	1	2	6	Moderate
Çalışkan et al. (2021) [19]	3	1	2	6	Moderate
Chandak (2024) [20]	2	1	2	5	Moderate
Chandukutty et al. (2017) [21]	3	1	2	6	Moderate
Gill et al. (2022) [22]	3	1	3	7	High
Koçkanat (2025) [23]	3	1	2	6	Moderate
Nazir et al. (2025) [24]	4	1	2	7	High
Ningthoujam et al. (2019) [25]	3	1	2	6	Moderate
Noor et al. (2023) [26]	3	1	2	6	Moderate
Qazi et al. (2009) [27]	2	1	2	5	Moderate
Rashmi et al. (2023) [28]	2	1	2	5	Moderate
Rouijel et al. (2024) [29]	3	1	2	6	Moderate
Vasconcellos et al. (2009) [12]	3	1	3	7	High

The included studies primarily evaluated awareness and knowledge regarding tooth avulsion and its emergency management among defined populations, including parents, school teachers, dental students, dental professionals, and emergency physicians. All studies employed structured or self-administered questionnaires [12-29], while one included a pre- and post-educational intervention design [30]. The majority of questionnaires were adapted to local languages and validated prior to use, ensuring cultural and linguistic relevance. It is summarized in Table 3.

**Table 3:** Summary characteristics of the included studies

Author(s) & Year	Study Design	Participants	Sample Size (n)	Country	Assessment Tool
Al-Asfour et al. (2008) [30]	Cross-sectional (with educational intervention)	Intermediate school teachers	74 (pre), 43 (post)	Kuwait	Structured interview / questionnaire (Arabic)
Al Barkhati et al. (2023) [13]	Cross-sectional	Emergency physicians	18	Oman	Standardized and validated questionnaire (English)
Al-Khalifa et al. (2022) [13,14] [15,16]	Cross-sectional	Intermediate school teachers	398	Saudi Arabia	Self-administered questionnaire (Arabic)
Al Sheeb et al. (2023) [15]	Cross-sectional	Parents of children attending dental appointments	400	Qatar	Self-administered questionnaire (Arabic)
Al-Huthaifi et al. (2025) [16]	Cross-sectional	Dental professionals	202	Yemen	Validated questionnaire (Arabic/English)
AlGhamdi et al. (2016) [13,14] [15–18]	Cross-sectional	Parents of children receiving dental care	274	Saudi Arabia	Self-administered questionnaire
Alyahya et al. (2018) [18]	Cross-sectional	Parents visiting dental specialty centers	554	Kuwait	Self-administered questionnaire (Arabic)
Çalışkan et al. (2021) [13,14] [15–19]	Cross-sectional	Emergency physicians (EPs)	100	Turkey	Close-ended (multiple-choice) questionnaire
Chandak (2024) [13,14] [15–20]	Cross-sectional	School teachers	146	India	Self-administered questionnaire (English & Marathi)
Chandukutty et al. (2017) [13,14] [15–21]	Cross-sectional	School teachers	303	India	Self-administered questionnaire
Gill et al. (2022) [13,14] [15–22]	Cross-sectional	Parents of school-going children	312	India	Online questionnaire (Google Form)
Koçkanat (2025) [13,14] [15–23]	Cross-sectional	Parents visiting a dental faculty	250	Turkey	Self-administered questionnaire (Turkish)
Nazir et al. (2025) [13,14] [15–24]	Cross-sectional	Elementary school teachers	610	Pakistan	Self-administered questionnaire
Ningthoujam et al. (2019) [13,14] [15–25]	Cross-sectional	Parents of school children (6-12 years)	777	India	Self-administered questionnaire (English & Manipuri)
Noor et al. (2023) [13,14] [15–26]	Cross-sectional	Undergraduate dental students (3rd & final year)	91	Pakistan	Questionnaire (close-ended questions)
Qazi et al. (2009) [13,14] [15–27]	Cross-sectional	Dentists, doctors, students, teachers, general public	377	Pakistan	Open-ended questionnaire form
Rashmi et al. (2023) [13,14] [15–28]	Cross-sectional	Parents of children (6-12 years) from rural/urban areas	400	India	Closed-ended questionnaire (English & Malayalam)
Rouijel et al. (2024) [13,14] [15–29]	Cross-sectional	Primary school teachers	202	Morocco	Self-administered questionnaire (French)
Vasconcellos et al. (2009) [13,14] [12,15–29]	Cross-sectional	General dental practitioners (GDPs)	264	Brazil	Multiple-choice questionnaire (21 items)

Awareness and Knowledge Among Dental Populations

Six studies assessed dental professionals and students. Awareness that an avulsed permanent tooth could be replanted was generally high, ranging from 87.6% among Yemeni dental professionals [16] to 66% among final-year dental students in Pakistan [26]. However, detailed knowledge of emergency protocols was inconsistent. Only 41.6% of Yemeni dentists recognized the critical replantation window (<30 minutes), and 40.1% identified HBSS as the ideal storage medium [16]. Among Brazilian general dentists, 37.1% recommended immediate replantation at the accident site, while 83.4% correctly identified milk or saline as suitable storage media [12]. In Pakistan, 45.8% of dentists suggested immediate replantation, but only 39.1% specified handling the tooth by the crown [27]. Knowledge of splinting duration and follow-up protocols was also suboptimal, with only 26.7% of Yemeni dentists correctly recommending 7-14 days of flexible splinting [16]. Educational exposure influenced outcomes; final-year students consistently outperformed junior students [26], and dentists with recent trauma training demonstrated higher procedural accuracy.

Awareness and Knowledge Among Non-dental Populations

Thirteen studies focused on non-dental groups, including parents, schoolteachers, and emergency physicians. Awareness levels were markedly lower. Only 23.2% of Indian parents knew an avulsed tooth could be replanted [28], and among schoolteachers, awareness ranged from 13% in Pakistan [24] to 79% in Saudi Arabia [14]. Willingness to attempt immediate replantation was minimal: 2% of Indian parents [22] and 13% of Pakistani teachers [24]. Knowledge of appropriate storage media was poor, with only 2.3-15.2% of parents identifying milk as suitable across studies. Emergency physicians in Oman and Turkey demonstrated moderate theoretical awareness (72% and 28%, respectively) but low confidence in performing replantation [13,19]. Educational interventions yielded significant improvements. For example, a brief training session for Kuwaiti teachers increased knowledge of the correct storage medium from 4% to 86% [30].

Knowledge of Immediate Replantation and First Aid

The proportion of respondents willing or able to attempt immediate replantation as first-aid ranged from 2% to 37% across studies. Only a small fraction of parents and teachers indicated they would replant the tooth on-site; for instance, 2% of Indian parents [22,28] and 13% of Pakistani teachers [24]. In contrast, 37.1% of Brazilian general dentists recommended replantation at the accident site [12], highlighting the professional gap between dental and lay populations.

Knowledge of Suitable Storage Media

Knowledge of appropriate storage media for avulsed teeth was inconsistent and generally low among non-dental participants. Across all studies, the percentage of respondents identifying milk as a suitable medium ranged from 2.3% to 48.4%, while only one study reported awareness of HBSS at 40.1% among Yemeni dentists [16]. Among teachers, the use of tissue paper, dry storage, or water was commonly reported [20,21,29], indicating widespread misconceptions regarding storage conditions.

Knowledge of the “Golden Period” for Replantation

Understanding of the optimal time frame for successful reimplantation was notably poor. Awareness of the critical replantation window (< 30 minutes) ranged from 14.5% to 63.9% across studies [16,28]. Only a minority of participants in non-dental groups recognized the need for urgent dental consultation; for example, 30% of Saudi teachers [14] and 22.4% of Turkish parents [23]. Even among dental professionals, only 41.6% correctly identified the 30-minute reimplantation period [16].

Knowledge of Proper Handling Techniques

Few studies specifically assessed handling methods, but those that did found considerable misconceptions. Approximately 45% of parents in India knew that the tooth should be handled by the crown rather than the root [28], while similar awareness among final-year dental students in Pakistan was 45% [26]. Mismanagement of handling techniques was frequent among laypersons, potentially jeopardizing the periodontal ligament and reducing reimplantation success.

Comparative Trends Between Dental and Non-dental Populations

Collectively, the data indicate that dental professionals and students exhibit higher theoretical awareness but limited procedural knowledge, whereas non-dental populations demonstrate critically low awareness and confidence. Across studies involving dental professionals and students, between 41% and 88% recognized that a permanent tooth can be replanted or identified the correct replantation window, yet only 36-79% selected an appropriate storage medium, and 37% recommended immediate replantation [12,16,26]. In contrast, studies assessing non-dental populations - including parents, teachers, and general public groups - reported markedly lower accuracy, with only 2-13% willing to attempt immediate replantation, 2-15% identifying milk as a suitable medium, and 14-33% recognizing the critical time window [18,21-25,28,29]. These patterns support the synthesized estimate that dental groups showed approximately 50% correct knowledge, while non-dental respondents averaged around 15-20%, reflecting a substantial preparedness gap. A mixed-population study from Pakistan by Qazi et al. [27] further demonstrated this disparity, reporting that, while 45.8% of dentists recommended immediate replantation, only 10.1% of the overall sample - particularly non-dental participants -were aware of the correct first-aid steps.

Impact of Educational Interventions

Educational programs demonstrated marked improvements in knowledge outcomes. The Kuwaiti study by Al-Asfour et al. [30] reported significant gains across all domains after a 30-minute instructional session, with knowledge of correct storage medium rising from 4% to 86% and understanding of time sensitivity from 1% to 74%. Similar findings were echoed in other intervention-based programs targeting teachers and medical staff, confirming that brief, focused training can yield substantial gains in awareness and readiness.

Regional and Demographic Differences

Geographical and demographic variations were evident. Studies from the Middle East (Qatar, Yemen, Saudi Arabia, Kuwait) generally reported moderate awareness with low protocol accuracy, while South Asian studies (India, Pakistan) indicated markedly poor parental and teacher knowledge. Gender, educational attainment, and prior exposure to dental trauma were significant predictors of higher knowledge scores in several studies [18,23-25,28]. Populations with previous training or dental education consistently demonstrated superior awareness, highlighting the cumulative effect of structured learning. A summary of the key results across all included studies is presented in Table 4.

**Table 4:** Detailed summary of key results and findings from the included studies

Author(s) & Year	Participant Group	Awareness that Reimplantation is Possible/Recommended	Knowledge of Immediate Replantation as First Aid	Knowledge of Suitable Storage Medium (e.g., Milk, Saline)	Knowledge of Urgent Professional Help / Replantation Time (<30 min)	Knowledge of Proper Handling (Hold by Crown)	Main Conclusion
Al-Asfour et al. (2008) [30]	School Teachers (Kuwait)	39% (pre); 97% (post)	Not Explicitly Reported	4% (pre); 86% (post) knew suitable medium	1% (pre); 74% (post) knew importance of extra-oral time	Not Explicitly Reported	A short educational lecture dramatically improved teachers' knowledge from low to an adequate level in all assessed domains.
Al Barkhati et al. (2023) [13]	Emergency Physicians (Oman)	72% (knew replantation is essential)	17% (felt comfortable replanting themselves)	61% (chose saline as suitable medium)	Not Explicitly Reported	0.78	While key knowledge points were relatively high, practical confidence was very low. Knowledge level was associated with higher clinical grade and previous dental education.
Al-Khalifa et al. 2022) [14]	School Teachers (Saudi Arabia)	79% (knew which teeth to replant)	5% (would replant immediately)	28% (knew milk/saliva)	30% (knew timing urgency for avulsion)	0.57	School teachers lack proper knowledge of dental emergency management, highlighting the need for educational training programs.
Al Sheeb et al. (2023) [15]	Parents (Qatar)	0.233	12.9% (of those aware) were willing to do it	48.4% (of those aware)	0.278	Not Reported	Parental knowledge in Qatar is critically low, highlighting an urgent need for accessible educational tools and public awareness campaigns.
Al-Huthaifi et al. (2025) [16]	Dental Professionals (Yemen)	87.6% (recognized tooth should be reinserted)	Implied by high awareness	40.1% (HBSS), 36.1% (Milk)	63.9% (acknowledged critical time period)	Not Explicitly Reported	Knowledge was moderate but inadequate, with significant gaps in time-sensitive and technical aspects of avulsion management according to IADT guidelines.
AlGhamdi et al. (2016) [17]	Parents (Saudi Arabia)	0.262	Not explicitly reported	11.7% (Milk)	33.9% (within 30 min)	Not Explicitly Reported	There is a significant lack of awareness among parents. Most (56.3%) would discard the avulsed tooth, indicating an urgent need for educational campaigns.
Alyahya et al. (2018) [18]	Parents (Kuwait)	0.359	27.8% (would attempt self-replantation)	15.2% (knew milk/child's mouth)	29.2% (knew to replant immediately)	0.69	Parents in Kuwait did not have adequate knowledge. Previous information on avulsion was the only significant predictor of good knowledge.
Çalışkan et al. (2021) [19]	Emergency Physicians (Turkey)	28% would replant a permanent tooth	Not directly asked for self-replantation	40% (Milk 28%, Saline 12%)	28% (knew replantation time should be <30 min)	0.68	EPs possessed inadequate knowledge, especially regarding replantation and storage media. Treatment of avulsion should be included in the medical curriculum.
Chandak (2024) [20]	School Teachers (India)	Not Explicitly Reported	Not explicitly reported	3.1% (Milk); 58.5% chose tissue paper	17% (knew within 24 hours)	0.27	Poor knowledge was observed among school teachers. There is a critical need for educational initiatives and dental camps to improve emergency preparedness.
Chandukutty et al. (2017) [21]	School Teachers (India)	0.465	22.8% (The tooth should be replaced immediately)	14.2% (Milk)	45.9% (within 30 min)	Not Explicitly Reported	Among the school teachers surveyed, there was significantly very low knowledge of emergency management of dental trauma.
Gill et al. (2022) [22]	Parents (India)	0.548	2% would attempt self-replantation	12.8% (Milk)	0.471	Not Reported	There is an inadequate understanding of TDI management among parents, leading to ineffective first-aid and a poor prognosis for avulsed teeth.
Koçkanat (2025) [23]	Parents (Turkey)	6% (would replant themselves)	6% (would replant themselves)	10.4% (Milk)	22.4% (within 20 min)	0.352	Parents do not have sufficient knowledge. University graduates had higher knowledge levels than those with lower education. Educational programs are needed.
Nazir et al. (2025) [24]	School Teachers (Pakistan)	13% (would reimplant)	13% (would reimplant)	13% (Milk)	67% (knew help needed within 30 min)	Not Explicitly Reported	Teachers had poor knowledge but a positive attitude toward learning. Targeted educational programs are needed to improve emergency preparedness.
Ningthoujam et al. (2019) [25]	Parents (India)	0.598	11.8% (would attempt self-replantation)	9.7% (Milk)	22.2% (knew to replant immediately)	Not Explicitly Reported	There is a small degree of awareness. A huge majority (96.1%) were interested in receiving more information, warranting urgent educational efforts.
Noor et al. (2023) [26]	Dental Students (Pakistan)	Implied by responses	66% (Final year students)	79% (Final year students knew milk/saline/HBSS)	Implied by responses	45% (Final year students)	The knowledge of undergraduate dental students was generally poor, indicating a need for more comprehensive training in the curriculum.
Qazi et al. (2009) [27]	Mixed Groups (Pakistan)	Not directly reported	10.1% (All respondents); 45.8% (Dentists only)	64% (Milk) & 20% (Saline) among dentists suggesting transport	Not directly reported	39.1% (of dentists suggesting replantation)	Non-dentists, including doctors, have insufficient knowledge. Dentists know more but need training on precise protocol details.
Rashmi et al. (2023) [28]	Parents (India)	23.2% (inferred)	2% would attempt self-replantation	2.3% (Milk); 41.5% had no knowledge	14.5% (within 30 min)	~45% (selected "crown")	Parental awareness of first-aid measures is inadequate across all demographics, but there is a strong positive attitude toward receiving information.
Rouijel et al. (2024) [29]	Primary School Teachers (Morocco)	23.9% (would reimplant)	23.9% (would perform immediate reimplantation)	6.8% (Milk); 47.6% used inadequate media (paper/compress)	Not Explicitly Reported	Not Explicitly Reported	There is a concerning lack of knowledge among school teachers. Educational programs are necessary to improve the management and prognosis of avulsed teeth.
Vasconcellos et al. (2009) [12]	General Dentists (Brazil)	Not directly reported	37.1% recommended it at the accident site	83.4% (Milk 44.7%, Saline 38.7%)	59.1% (seek dentist immediately)	Not Reported	Brazilian GDPs demonstrated good knowledge in several areas but performed poorly regarding the critical step of immediate replantation.

Discussion

The findings of this systematic review illustrate the persistent global shortfall in knowledge and preparedness surrounding the emergency management of avulsed permanent teeth, particularly regarding timely reimplantation and appropriate transport media. Among dental professionals and students, awareness that a knocked-out tooth should ideally be reimplanted is relatively high; for example, a study in Yemen found that 87.6% of dental respondents recognized the necessity of reinsertion [16]. However, only 40.1% knew the ideal transport medium, and only 63.9% acknowledged the critical extra-oral time window for successful replantation [16]. Similarly, a study of interns in Pakistan found that 71% were aware of initial management of avulsed teeth, but only 49% knew the correct transport medium, and 43% knew the critical time window [9]. These findings suggest that, while the concept of avulsion as a dental emergency is widely accepted within dental training, the detailed protocol-based knowledge (e.g., storage media, timing, splinting, follow-up) remains suboptimal.

In non-dental stakeholder groups - such as school teachers, first-aid providers, and parents - the situation is even more concerning. For example, a study of primary school teachers in Morocco found that only 23.9% indicated that they would attempt immediate replantation and only 6.8% would place the tooth in milk (a recognized transport medium) [29]. A survey of teachers in Saudi Arabia reported that only about 30% had first-aid training, and only 11% of that training addressed dental traumas; the overall knowledge of avulsion management was inadequate [14]. Among parents, one study found that 76.8% did not know that a child with an avulsed tooth could still chew and smile normally after reimplantation, and only 2.3% selected milk as the transport medium [28]. These deficits point to a major gap in first-response preparedness, which is crucial because the prognosis of an avulsed tooth is highly time-dependent and reliant on appropriate immediate management.

Understanding the key variables that influence prognosis helps explain why these knowledge gaps matter. The literature emphasizes that extra-alveolar time and choice of storage/transport medium are among the most important determinants of success. A systematic review with meta-analysis on storage media found that whilst HBSS remains the ideal physiologic medium, milk, saliva, and other accessible fluids also offer significantly better outcomes than dry storage [31]. Translating those findings into practice, a scoping review noted that immediate reimplantation (within 30 minutes) is the gold standard, but delayed reimplantation is far more common and considerably reduces survival rates [2]. Thus, if first-aid responders or bystanders fail to either reinsert or store the tooth appropriately, the long-term prognosis is compromised.

The disparity between knowledge levels among dental versus non-dental groups may reflect differences in training, exposure, and institutional emphasis. Dental students and professionals receive theoretical instruction - though the consistency and depth vary - while teachers, sports coaches, and parents often have little or no formal education in dental trauma management. In a study among emergency physicians, nearly half reported they did not know what to do when faced with an avulsed tooth; only a minority felt confident in managing the situation [19]. This suggests that many of the potential first-point contacts for trauma victims are under-prepared to act appropriately, which places a greater burden on dental professionals and reduces the opportunity for optimal outcomes.

The implications of these findings are broad. At the curricular level, dental education programs may need to place greater emphasis not just on recognizing avulsion but on the full management algorithm - including handling the tooth, selecting the medium, timing, referral, splinting, and follow-up. A study among dentists in Riyadh, Saudi Arabia, found moderate knowledge (mean score: 5.94±1.57 out of a maximum of 10), with particularly low awareness concerning ideal transport medium (24.1%) and duration of follow-up (15.6%) [32]. For non-dental groups, simple, practical training programs (e.g., a 30-minute lecture for teachers) have shown marked improvements in knowledge - for example, improvement from 39% to 97% general knowledge in one such intervention [30]. Thus, public health efforts should consider designing accessible, hands-on training modules for school staff, sports coaches, and other first-aid providers, emphasizing clear, actionable steps: retrieve the tooth by the crown, rinse briefly if dirty, reinsert if possible, or store in milk/physiological medium, and refer immediately.

There are, however, limitations in the body of evidence that warrant consideration. Many studies use self-administered questionnaires, which assess declared knowledge rather than observed behavior; this may overestimate actual competence in real-life emergencies. There is also heterogeneity in study designs, populations, questionnaires, and definitions of “adequate knowledge,” making cross-study comparisons difficult. The majority of evidence comes from certain regions (the Middle East, South Asia) and may not reflect global diversity in resources or trauma incidence. Additionally, few studies evaluate long-term outcomes (e.g., tooth survival following reimplantation), focusing instead on knowledge rather than procedural follow-through or success rates. For example, while a case report illustrated successful reimplantation after storing a tooth in milk for five hours, which is beyond the typical recommended time windows, such cases are anecdotal and cannot substitute for systematic longitudinal data [33]. Systematic evidence indicates that dental trauma can alter orthodontic parameters and treatment planning, underscoring the importance of timely management and longitudinal follow‑up [34]. Furthermore, this review was limited to studies published in English, as specified in the eligibility criteria. This may have introduced a language bias by excluding potentially relevant evidence published in other languages, which could affect the comprehensiveness of the findings. Future research should incorporate validated, standardized instruments, larger and more geographically diverse samples, and outcome-based assessments of management effectiveness rather than solely knowledge. Qualitative work to explore barriers (e.g., lack of resources, fear of liability, logistic hurdles) to correct management would also enrich understanding. Therefore, while there is growing recognition that tooth avulsion is a dental emergency among dental professionals, critical gaps remain, particularly in detailed protocol knowledge and first-response readiness among non-dental populations. As the prognosis of an avulsed tooth is heavily time-sensitive and management-dependent, addressing these gaps is essential. Targeted educational strategies at the undergraduate, professional, and community levels have the potential to improve outcomes and reduce the long-term burden of traumatic dental injuries.

## Conclusions

This systematic review highlights a significant global deficiency in awareness and knowledge regarding the emergency management of tooth avulsion and subsequent reimplantation. Despite clear evidence supporting the benefits of immediate and proper management in improving the prognosis of avulsed teeth, both dental and non-dental populations demonstrate inadequate understanding of the essential first-aid steps. While dental professionals and students generally possess higher theoretical knowledge, many lack sufficient familiarity with time-sensitive clinical protocols such as the optimal replantation window, suitable storage media, and correct handling techniques. Conversely, non-dental individuals - including parents, teachers, and medical professionals - show limited awareness of the possibility of reimplantation or its urgency. These findings underscore the urgent need for targeted educational interventions. Integrating dental trauma management modules into school health programs, undergraduate medical and dental curricula, and community awareness campaigns could substantially enhance public preparedness. Furthermore, professional continuing education should emphasize adherence to the IADT guidelines to ensure evidence-based practice. In conclusion, improving the global knowledge base on tooth reimplantation after avulsion requires coordinated efforts across educational, healthcare, and community sectors. Early recognition, prompt action, and proper referral can dramatically influence the prognosis of an avulsed tooth, reinforcing the importance of widespread, practical education on this preventable dental emergency.

## Appendices

**Table 5:** Search strategies for the included studies

Database/Register	Search String	Records
PubMed/MEDLINE	("Tooth Avulsion"[MeSH Terms] OR "tooth avulsion"[All Fields] OR "avulsed tooth"[All Fields] OR "dental avulsion"[All Fields]) AND ("Tooth Replantation"[MeSH Terms] OR "tooth reimplantation"[All Fields] OR replantation[All Fields]) AND ("Knowledge"[MeSH Terms] OR "Awareness"[MeSH Terms] OR knowledge[All Fields] OR awareness[All Fields] OR attitude[All Fields] OR education[All Fields])	n = 2459
ScienceDirect	("tooth avulsion" OR "avulsed tooth" OR "dental trauma") AND ("tooth reimplantation" OR replantation) AND (knowledge OR awareness OR attitude OR education)	n = 3040
Cochrane Library	("tooth avulsion" OR "avulsed tooth") AND ("tooth reimplantation" OR replantation) AND (knowledge OR awareness OR education)	n = 3
Google Scholar	("tooth avulsion" AND "tooth reimplantation" AND (knowledge OR awareness OR education))	n = 2631
